# Antibodies against immunogenic epitopes with high sequence identity to SARS-CoV-2 in patients with autoimmune dermatomyositis

**DOI:** 10.1136/annrheumdis-2020-217522

**Published:** 2020-05-22

**Authors:** Spyridon Megremis, Thomas D J Walker, Xiaotong He, William E R Ollier, Hector Chinoy, Lynne Hampson, Ian Hampson, Janine A Lamb

**Affiliations:** 1 Division of Evolution and Genomic Sciences, The University of Manchester, Manchester, UK; 2 Division of Cancer Sciences, The University of Manchester, Manchester, UK; 3 Division of Population Health, Health Services Research and Primary Care, The University of Manchester, Manchester, Manchester, UK; 4 Centre for Bioscience, Faculty of Science and Engineering, Manchester Metropolitan University, Manchester, UK; 5 National Institute for Health Research Manchester Biomedical Research Centre, Manchester University NHS Foundation Trust, The University of Manchester, Manchester, UK; 6 Department of Rheumatology, Manchester Academic Health Science Centre, Salford Royal NHS Foundation Trust, Salford, Salford, UK

**Keywords:** autoantibodies, dermatomyositis, autoimmune diseases

Idiopathic inflammatory myopathies (IIMs) are rare, heterogeneous, autoimmune musculoskeletal diseases, characterised clinically by muscle weakness. Extramuscular involvement includes the skin, respiratory and cardiovascular systems. Genetic and environmental factors contribute to IIM susceptibility, and viral or bacterial infection may contribute to disease pathogenesis.

Both the innate and adaptive immune systems are important in IIM pathology. Two-thirds of affected individuals have known myositis-specific or associated autoantibodies, often linked to particular clinical features,^1^ and directed against proteins involved in key intracellular processes. Interferon pathways are differentially activated in clinical subtypes of myositis^2^; this interferon response is critical to protect the host against viral infection and modulate the antiviral immune response.

We recently used a high-throughput approach combining disease-specific immunoglobulin epitope signature enrichment and antigen identification from the total microbial ‘exposome’ (including viruses, bacteria, archaea and fungi) and human proteins.^3^ We applied this serum antibody repertoire analysis pipeline to investigate the microbial and autoantigen antibody repertoire accumulated throughout life in 20 adult-onset dermatomyositis patients seropositive for TIF1γ (TRIM33) autoantibodies, compared with 20 age-matched and gender-matched healthy controls.^3^

Human coronaviruses are associated with the common cold, but can lead to fatal inflammatory responses and acute lung injury. Emergence of a novel coronavirus has caused a recent global pandemic of severe acute respiratory syndrome (SARS) in humans (COVID-19).^4^ Whole genome phylogenetic analyses suggest that the COVID-19 coronavirus SARS-CoV-2 shares high sequence similarity with bat coronaviruses and the host reservoir is bats.^4^

**Figure 1:**
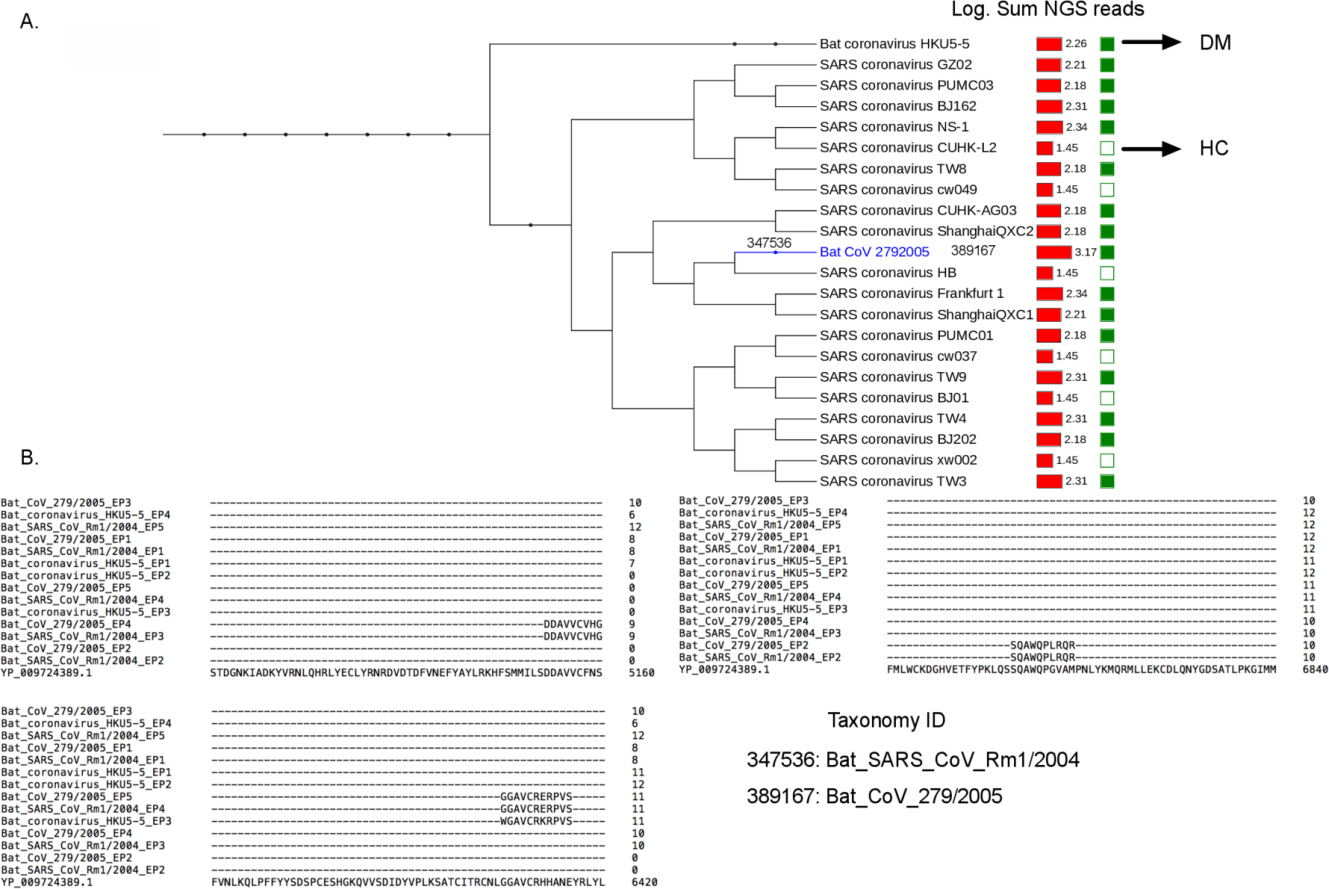
Coronavirus species and epitopes. (A) Taxonomy tree of coronavirus species identified in the study. The total number of next-generation sequencing (NGS) reads per species is visualised as red bar plots. Green squares: presence only in dermatomyositis (DM), squares with no filling: presence only in healthy controls (HC). (B) Partial alignments of the three bat coronavirus epitopes which are shared with SARS-CoV-2. SARS-CoV-2, severe acute respiratory syndrome coronavirus-2.

Due to the current coronavirus pandemic, here, we focused our analysis on epitopes mapping to the coronaviridae family. In dermatomyositis patients^3^, we identified enrichment of immunogenic linear epitopes (minimum 10 consecutive amino acids) mapping to 20 coronaviridae species, including 10 discrete epitopes mapping to three bat-coronavirus species. To investigate whether these 10 bat-coronavirus epitopes share sequence identity with human SARS-CoV-2, we carried out local alignment of the identified epitope sequences and the orf1ab polyprotein of SARS-CoV-2 (NCBI RefSeq: YP_009724389.1), and identified six distinct epitopes with high sequence identity (table 1). The epitopes were further queried against the database of non-redundant protein sequences (NCBI Blastp suite). Three linear epitopes of six amino acid length were highly specific for SARS-CoV-2 (table 1, figure 1). These epitopes map to SARS-CoV-2 2'-O-ribose methyltransferase, RNA-dependent RNA polymerase and 3'-to-5' exonuclease proteins. All three epitopes show extremely high conservation among currently available SARS-CoV-2 polyprotein sequences from the NCBI database (NCBI Multiple Alignment).

**Table 1:** Immunogenic epitopes enriched in dermatomyositis patients with sequence identity between bat coronavirus and SARS-CoV-2

Bat coronavirus strain/epitope	Immunogenic epitope	Human SARS-CoV-2 sequence identity (YP_009724389.1)	Length	Start-end (AA)	RefSeq protein	Protein
BtCoV/279/2005_EP1	VKGECPVMAPRR	GECP*	4	268–271	YP_009725298.1	Non-structural protein 2
BtCoV/279/2005_EP2	SQAWQPLRQR	SQAWQP†	6	6800–6805	YP_009725311.1	2'-O-ribose methyltransferase
		LRQ*	3	6883–6885	YP_009725311.1	2'-O-ribose methyltransferase
BtCoV/279/2005_EP4	DDAVVCVHGL	DDAVVC†	6	5152–5157	YP_009725307.1	RNA-dependent RNA polymerase
BtCoV/279/2005_EP5	GGAVCRERPVS	GGAVCR†	6	6405–6410	YP_009725309.1	3'-to-5' exonuclease
Bat_SARS_CoV_Rm1/2004_EP1	VKGECPVMAPRR	GECP*	4	268–271	YP_009725298.1	Non-structural protein 2
Bat_SARS_CoV_Rm1/2004_EP2	SQAWQPLRQR	SQAWQP†	6	6800–6805	YP_009725311.1	2'-O-ribose methyltransferase
		LRQ*	3	6883–6885	YP_009725311.1	2'-O-ribose methyltransferase
Bat_SARS_CoV_Rm1/2004_EP3	DDAVVCVHGL	DDAVVC†	6	5152–5157	YP_009725307.1	RNA-dependent RNA polymerase
Bat_SARS_CoV_Rm1/2004_EP4	GGAVCRERPVS	GGAVCR†	6	6405–6410	YP_009725309.1	3'-to-5' exonuclease
Bat_coronavirus_HKU5-5_EP2	SAGCFVGLPIAG	AGCFV‡	4	5232–5236	YP_009725307.1	RNA-dependent RNA polymerase
Bat_coronavirus_HKU5-5_EP3	WGAVCRKRPVS	GAVCR‡	5	6406–6410	YP_009725309.1	3'-to-5' exonuclease

Disease-specific immunogenic epitopes identified against which immunoglobulins were raised. Cross-species alignment carried out using Clustal-O and CLC Genomics Workbench 12.

*Blastp: not available

†Blastp: High specificity for SARS-CoV-2

‡Blastp: Low specificity for SARS-CoV-2

AA, amino acid; SARS-CoV-2, severe acute respiratory syndrome coronavirus-2.

We subsequently investigated whether these epitopes have been experimentally identified as B cell and T cell immunogenic epitopes from studies of epidemic-causing virus SARS-CoV, or computationally predicted from SARS-CoV-2. Epitope ‘DDAVVC’ in the RNA-dependent RNA polymerase protein is a highly ranked CD8 T cell predicted epitope identified from the Immune Epitope Database and Analysis Resource, showing HLA-A*01:01 restriction.^5^

The coronavirus genome encodes four structural proteins; spike, nucleocapsid, membrane and envelope proteins. SARS-CoV-2 spike glycoproteins promote cell entry through attachment to the host ACE 2 receptor, and subsequent fusion between viral and host cell membranes to facilitate viral entry,^6^ and are the main target of antibodies. Here, we report identification of three immunogenic linear epitopes with high sequence identity to SARS-CoV-2 proteins in patients with autoimmune dermatomyositis, including ‘DDAVVC’ in the RNA-dependent RNA polymerase protein previously predicted as a CD8 T cell epitope,^5^ in keeping with T cell antigen presentation derived from processing both structural and non-structural proteins. The identification of immunogenic coronavirus epitopes with high sequence identity may indicate SARS-CoV-2 targets for vaccine development against COVID-19. Latent exposure to the coronaviridae family might contribute to musculoskeletal autoimmune disease development, as illustrated by a recent report of myositis in a patient with COVID-19.^7^
